#  Corrigendum: The non-canonical mitochondrial inner membrane presequence translocase of trypanosomatids contains two essential rhomboid-like proteins

**DOI:** 10.1038/ncomms16123

**Published:** 2017-06-30

**Authors:** Anke Harsman, Silke Oeljeklaus, Christoph Wenger, Jonathan L. Huot, Bettina Warscheid, André Schneider

Nature Communications 7: Article number:13707 ; DOI: 10.1038/ncomms13707 (2016); Published: 12 19 2016; Updated: 06 30 2017

The original version of this Article contained an error in the gene ID for TimRhom I, cited in the Methods, which specified an unrelated gene. The correct gene ID for TimRhom I is Tb927.9.8260. This has now been corrected in both the PDF and HTML versions of the Article.

