# Supplementary material for: The non-canonical mitochondrial inner membrane presequence translocase of trypanosomatids contains two essential rhomboid-like proteins
Source: Nat Commun. 2016 Dec 19;7:13707. doi: 10.1038/ncomms13707 (PMC5187411; doi:10.1038/ncomms13707)
Supplement: Supplementary Information — Supplementary Figures 1-5, Supplementary Note 1, Supplementary References. [file ncomms13707-s1.pdf]

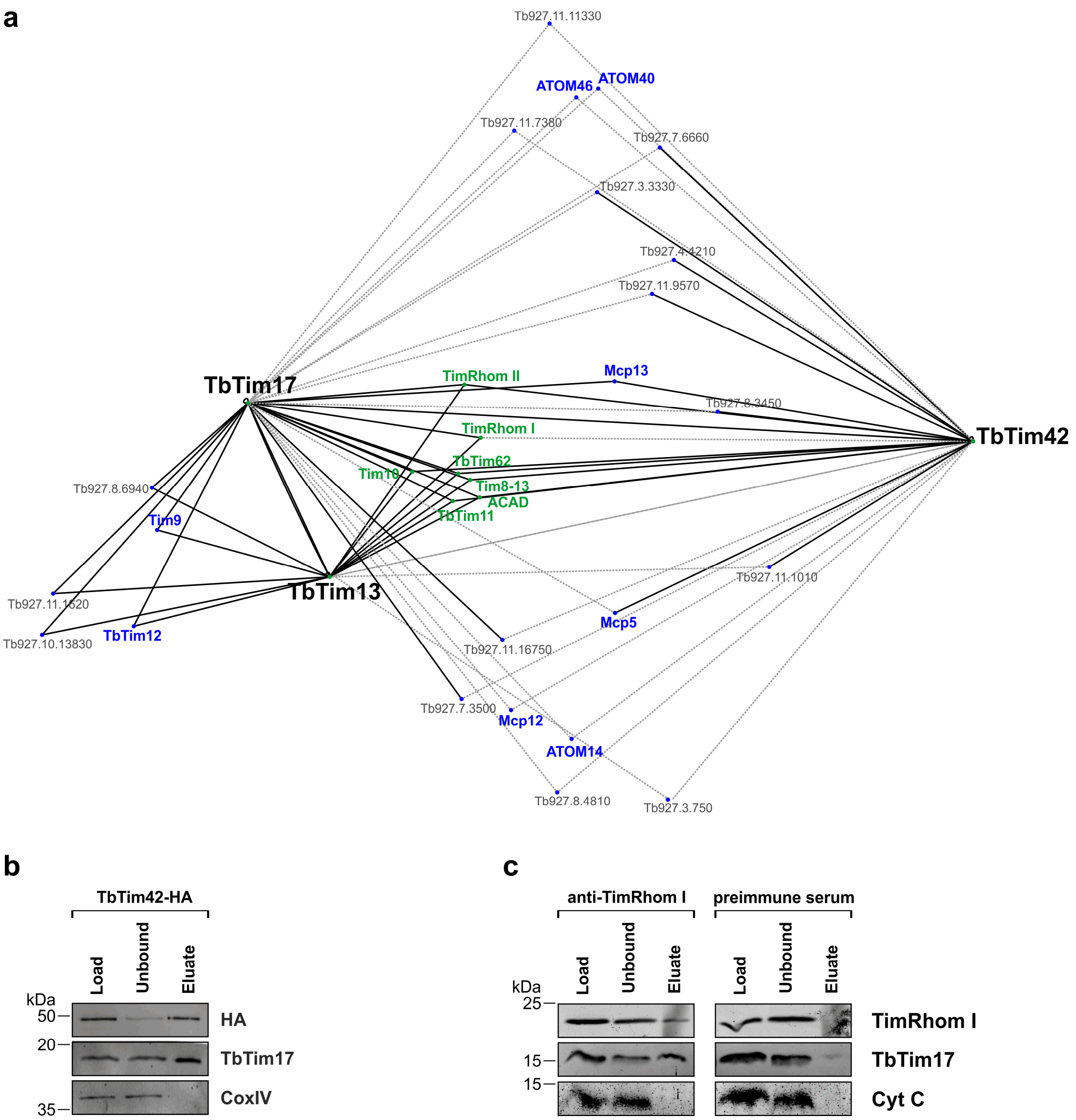

Supplementary Figure 1. (a) Network view of the overlap between the three sets of SILAC-IPs. Network was produced using Cytoscape[1], with the connecting lines corresponding to the detected enrichment factors. All proteins that were enriched with the bait in two (blue) or all three (green) SILAC-IPs are shown. Enrichment factors of > 2-fold and > 5-fold are indicated by grey dashed and black solid lines, respectively. **(b)** IPs of digitonin-solubilized mitochondrial extracts from cells expressing HA-tagged TbTim42. 10% of the lysate (load) and the unbound fraction as well as 100% of the Eluate were separated by SDS PAGE and subjected to immunoblotting. The blots were probed for HA, TbTim17 and CoxIV. **(c)** IPs of digitonin-solubilized mitochondrial extracts from wildtype *T. brucei* cells. Immunoprecipitation of TimRhom I using anti-TimRhom I antibodies coupled to Protein G-Sepharose.

10% of the lysates (load) and the unbound fractions as well as 100% of the Eluates were separated by SDS PAGE and subjected to immunoblotting. The blots were probed for TimRhom I, TbTim17 and Cyt C.

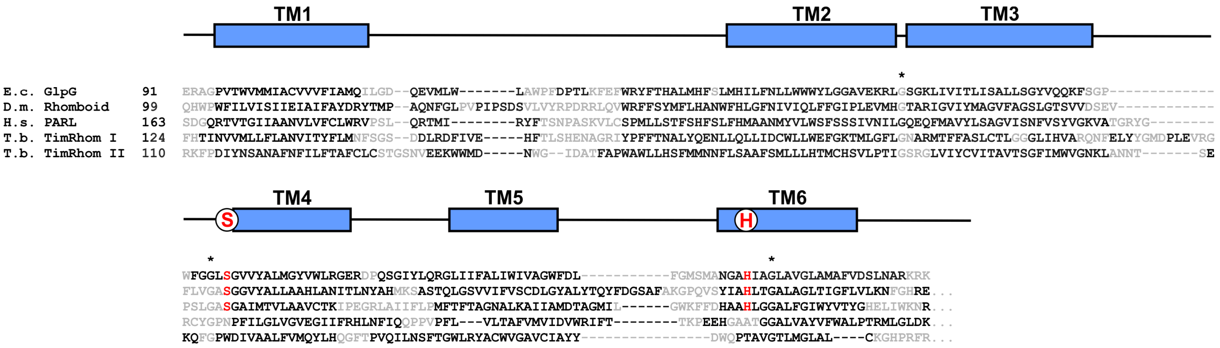

Supplementary Figure 2. Structural alignment of TimRhom I and TimRhom II with known Rhomboid proteases. The members of the rhomboid family of proteins are known to show only low sequence similarity. The depicted alignment of the well characterized rhomboid-like proteins GlpG from *Escherichia coli*, Rhomboid from *Drosophila melanogaster*, mitochondrial presenilins-associated rhomboid-like protein (PARL) from *Homo sapiens* with TimRhom I and TimRhom II from *T. brucei* was produced using PROMALS3D without user-defined constraints[2]. Residues marked with * are conserved in all five depicted proteins. Black letters indicate predicted helical structure. The respective transmembrane helices (TM1-TM6) and the Ser-His catalytic dyad of active rhomboid proteases are indicated.

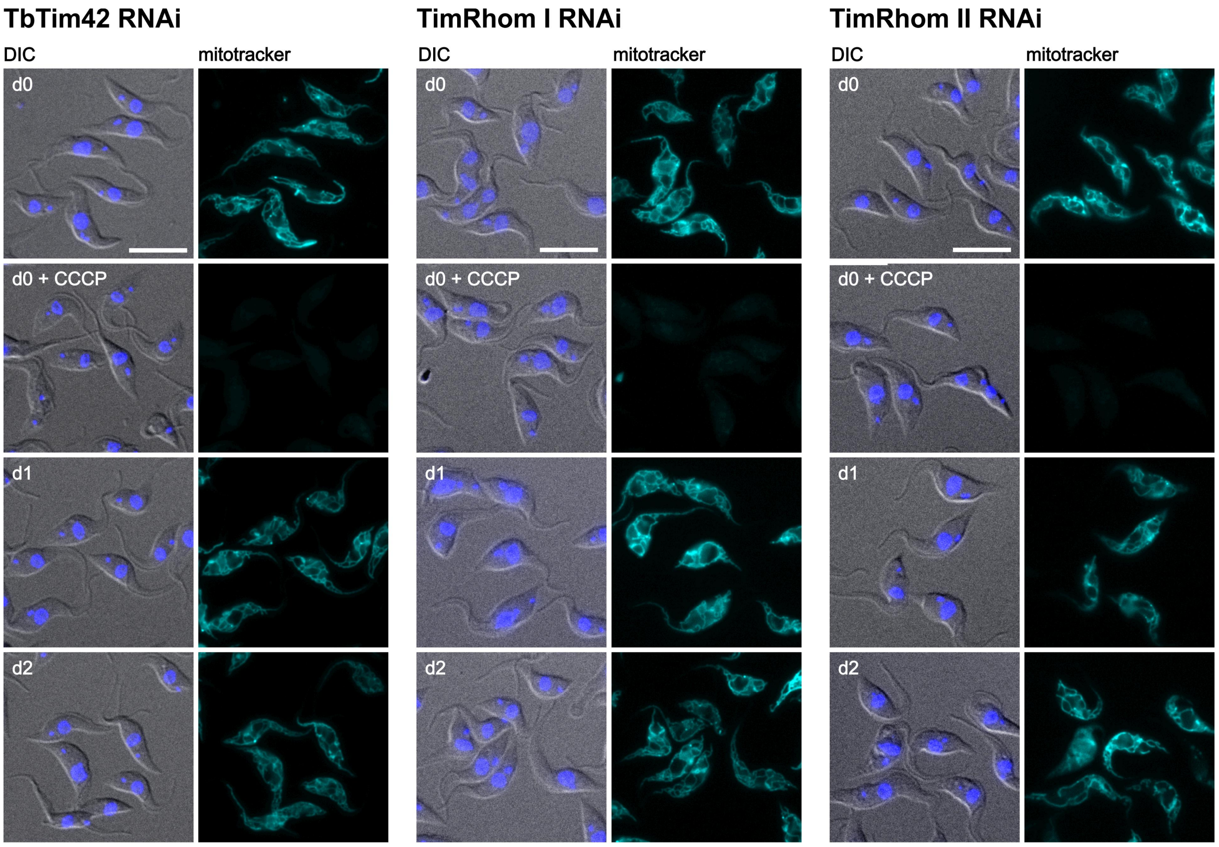

Supplementary Figure 3. RNAi-mediated ablation of TIM subunits does not abolish the mitochondrial membrane potential at early time points of induction. Differential interference contrast microscopy (DIC) and corresponding Mitotracker staining of the indicated uninduced (0d) and induced RNAi cell lines. Time of tetracycline induction in days is indicated. Uninduced cells treated with 40 µM of carbonyl cyanide m-chlorophenylhydrazone (CCCP) serve as negative controls for cells whose mitochondria have lost the membrane potential. Bar, 10 µm.

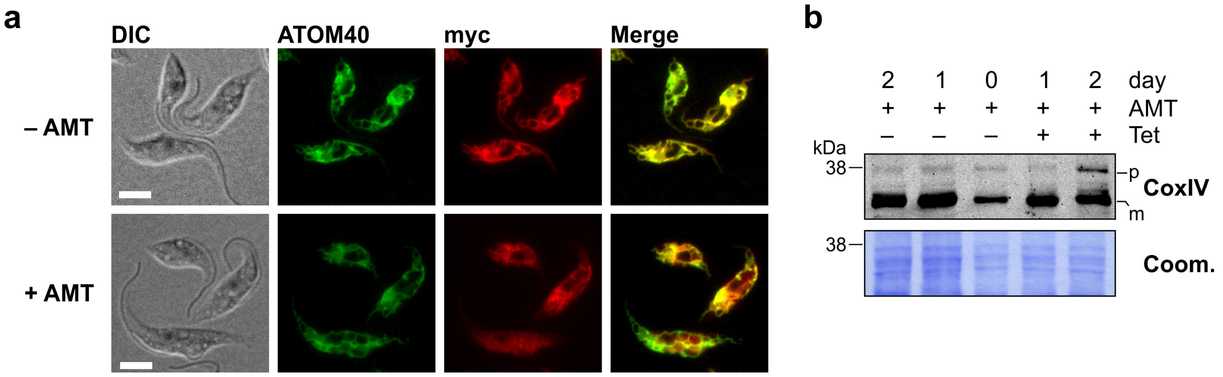

Supplementary Figure 4. The AMT-dependent import intermediate blocks further mitochondrial protein import. (a) IF analysis of cells expressing the myc-tagged chimeric precursor protein (LDH-DHFR-myc). Cells grown in the absence and presence of AMT were analyzed. ATOM40 serves as a mitochondrial marker. The differential interference contrast (DIC) image demonstrates integrity of the cells. Bar, 5 µm. **(b)** Cells allowing Tet-inducible expression of LDH-DHFR-myc where grown in the presence of AMT and the absence and presence of Tet for the indicated time period. Whole cell extracts (2.5Ã10^{6} cell equivalents) were separated by SDS-PAGE and the resulting immunoblot was probed for CoxIV. The positions of precursor (p) and mature (m) forms of CoxIV are indicated. The Coomassie-stained gel serves as loading control.

Full scans for Figure 1

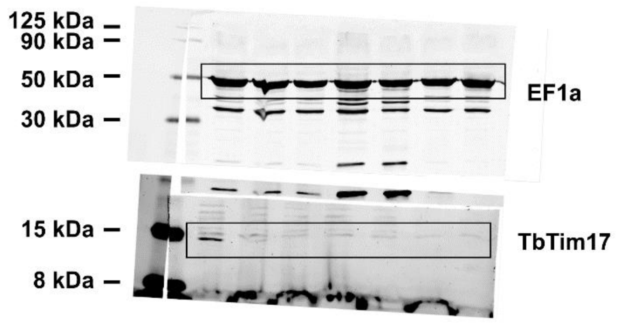

Licor - Chameleon Duo (P/N 928-60000)

Full scans for Figure 3a - top panel

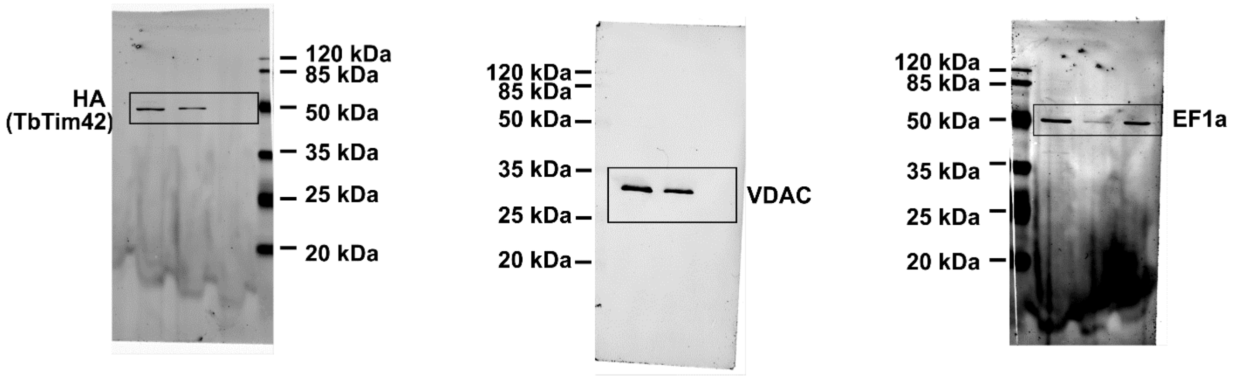

Thermo Scientific - Prestained protein molecular weight marker

Full scans for Figure 3a - middle panel

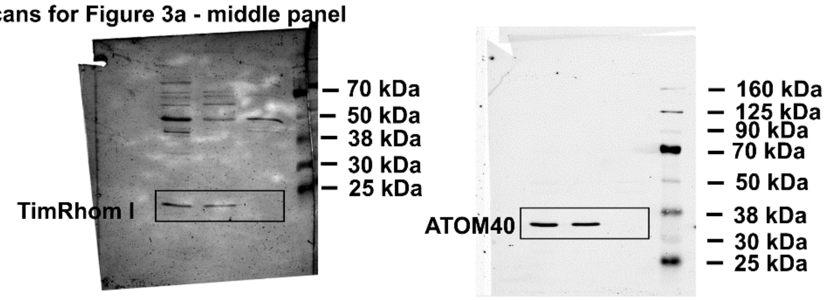

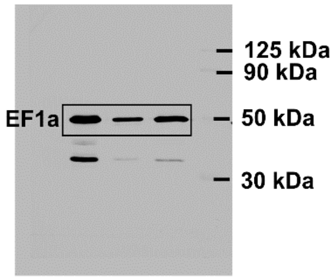

Licor - Chameleon Duo (P/N 928-60000)

Full scans for Figure 3a - bottom panel

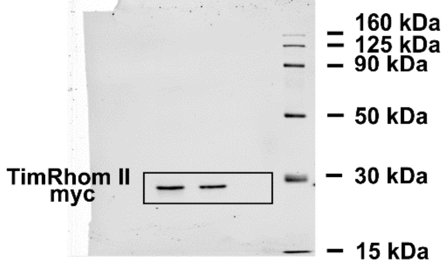

Western blot from the same samples as in middle panel, thus same ATOM40 and EF1a controls apply (see above)

Licor - Chamaeleon Duo (P/N 928-60000)

Full scans for Figure 3c - top panel

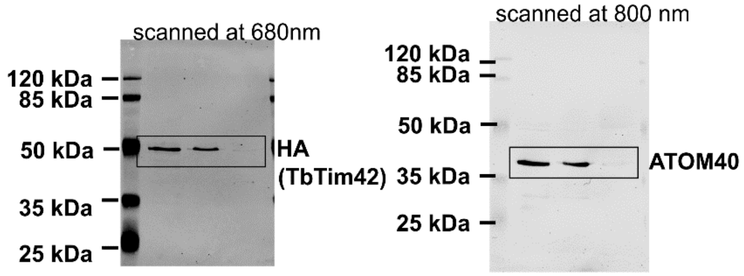

Thermo Scientific - Prestained protein molecular weight marker

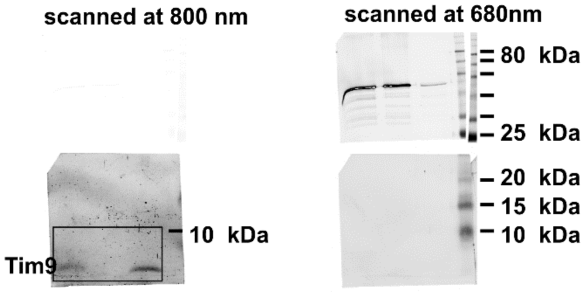

same blot scanned at different intensity to visualize marker

NEB - Prestained Protein Ladder, Broad Range (10-230 kDa)

Full scans for Figure 3c - middle panel

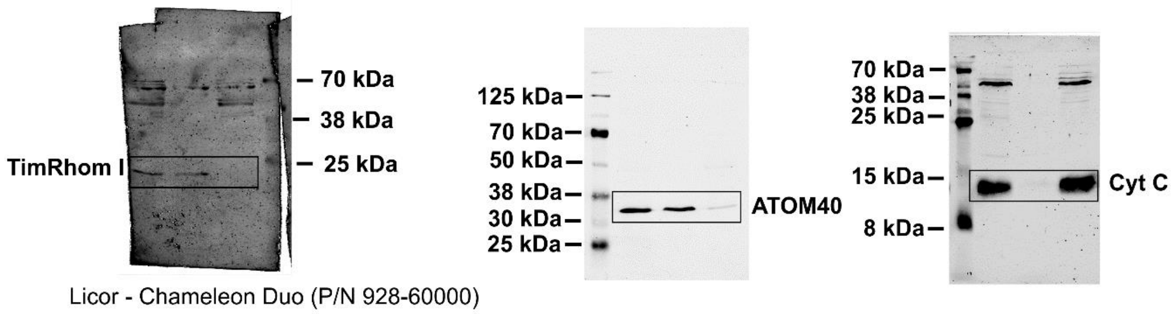

Licor - Chameleon Duo (P/N 928-60000)

Full scans for Figure 3c - bottom panel

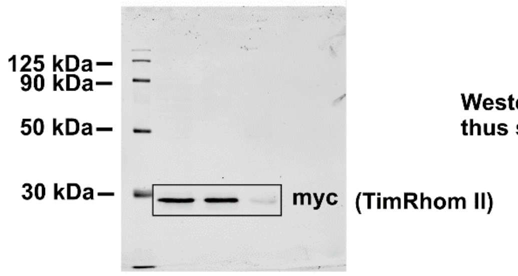

Western blot from the same samples as in middle panel, thus same ATOM40 and EF1a controls apply (see above)

Licor - Chameleon Duo (P/N 928-60000)

Full scans for Figure 4a - Northern blots of RNAi cell lines

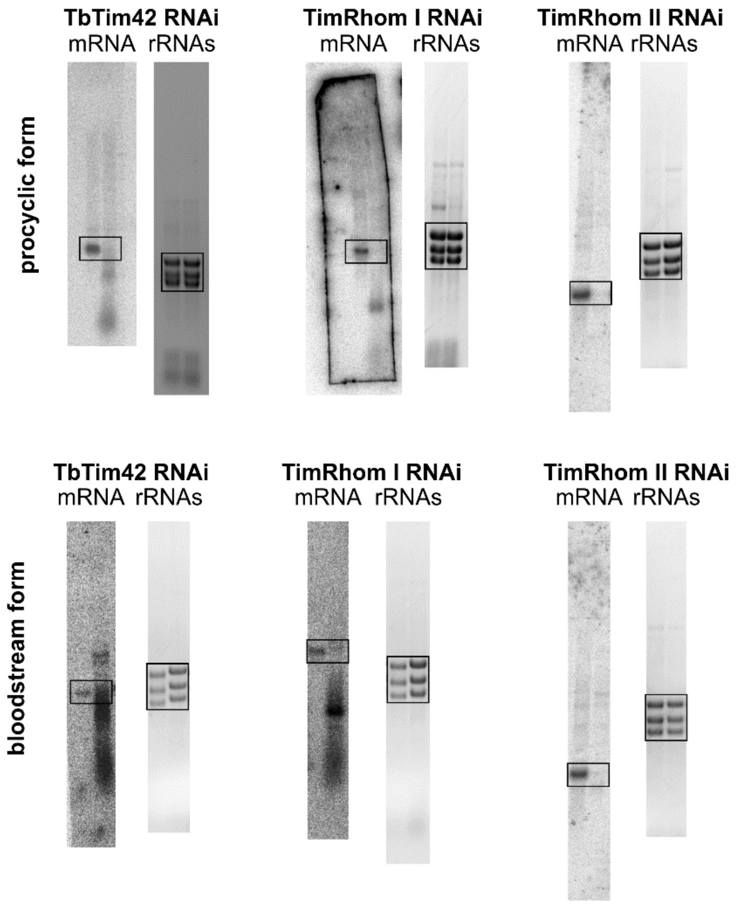

mRNA = Northern blot (autoradiography)
rRNAs = EtBr staining of total RNAs

Full scans for Figure 4b left panel - TbTim42 RNAi

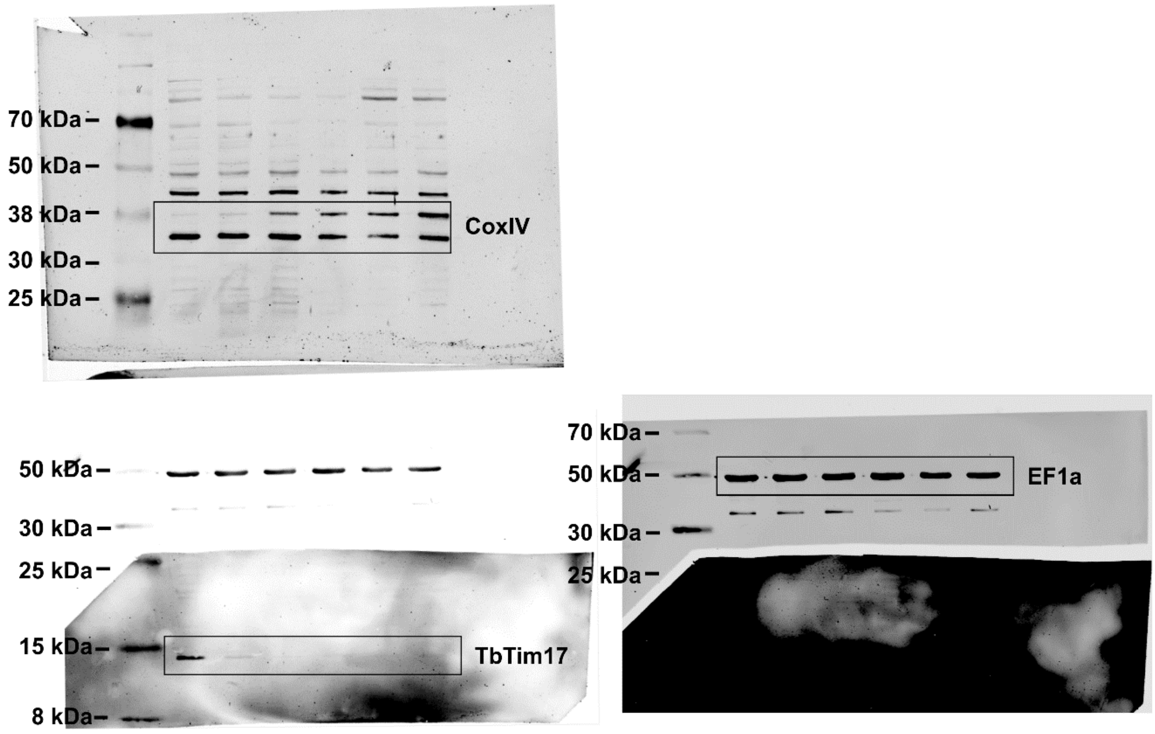

same blot scanned at different intensity

Licor - Chameleon Duo (P/N 928-60000)

Full scans for Figure 4b middle panel - TimRhom I RNAi

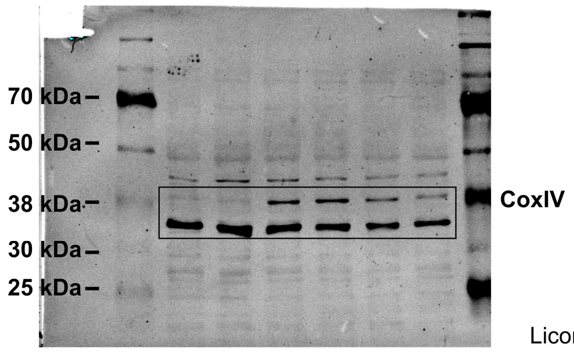

Licor - Chameleon Duo (P/N 928-60000)

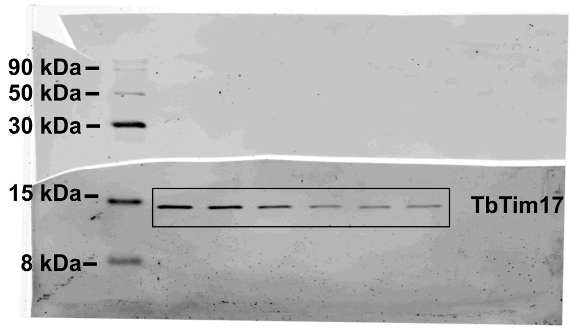

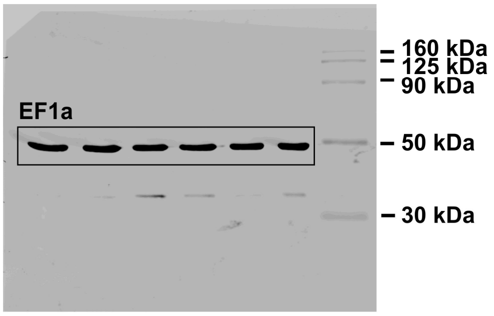

Full scans for Figure 4b right panel - TimRhom II RNAi

scanned 800 nm

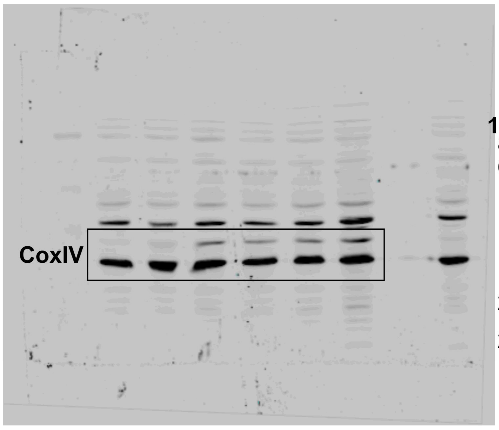

scanned 680 + 800 nm (to visualize marker)

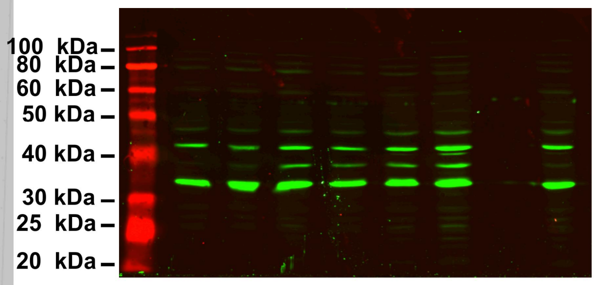

scanned 680 nm

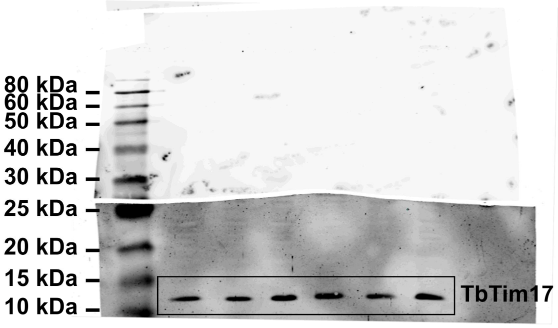

scanned 680 nm

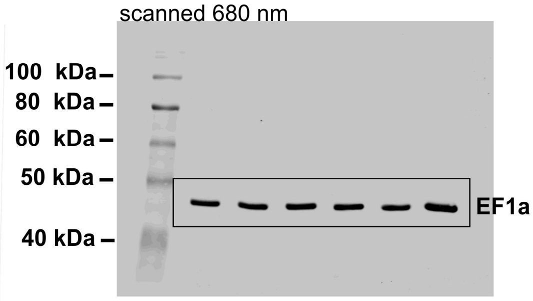

NEB - Prestained Protein Ladder, Broad Range (10-230 kDa)

Full scans for Figure 5c

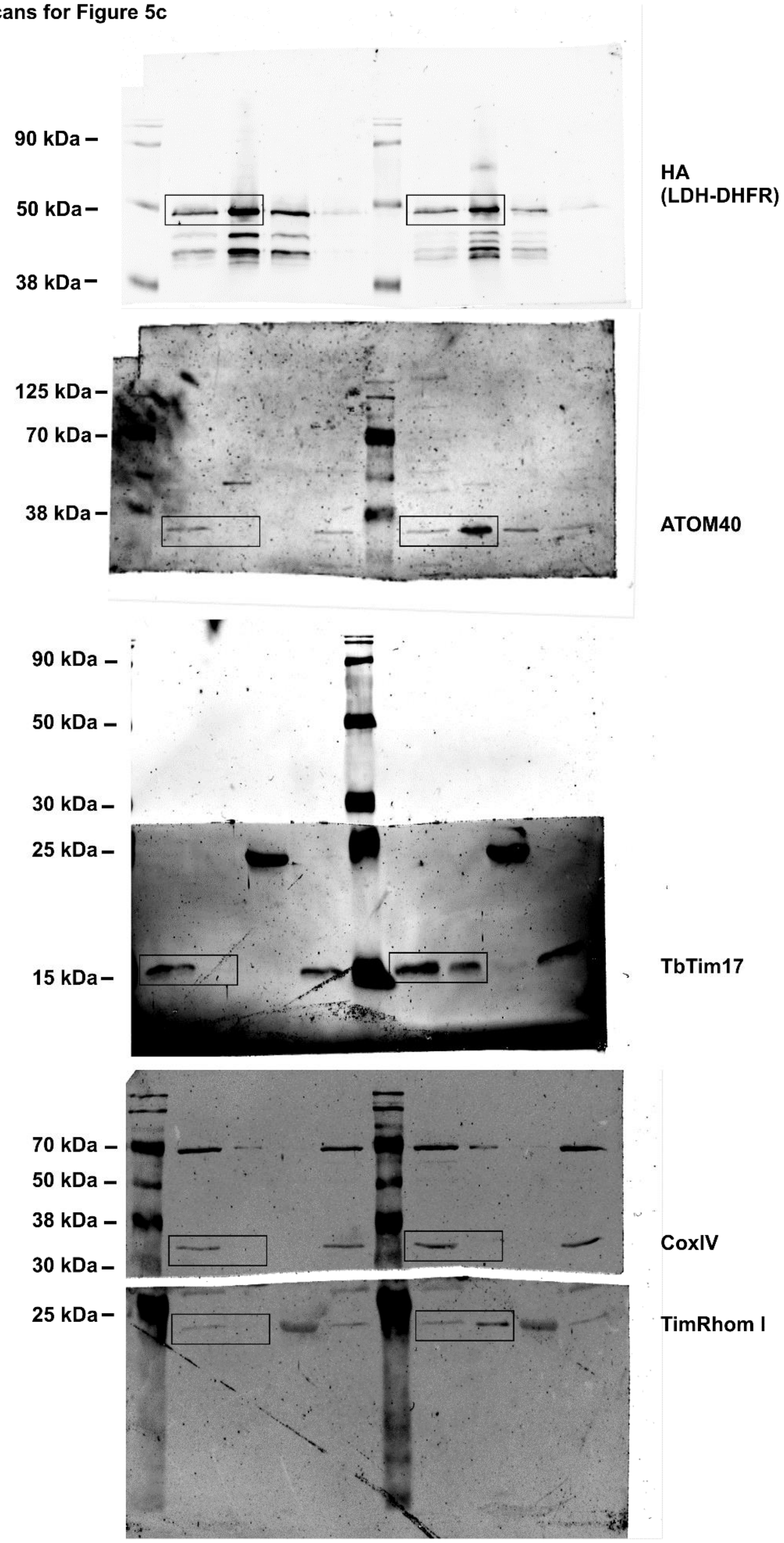

Licor - Chamaeleon Duo (P/N 928-60000)

Full scans for Figure 6b

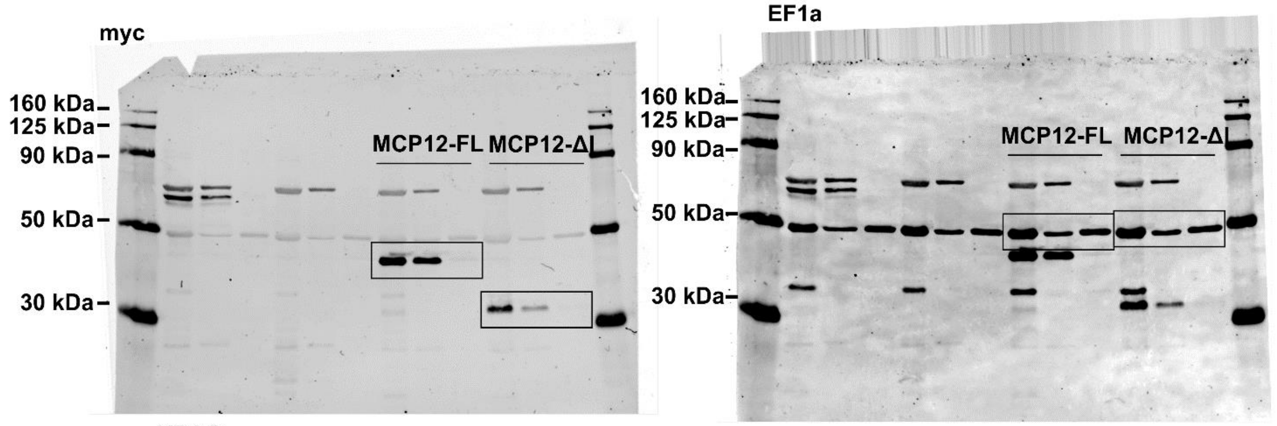

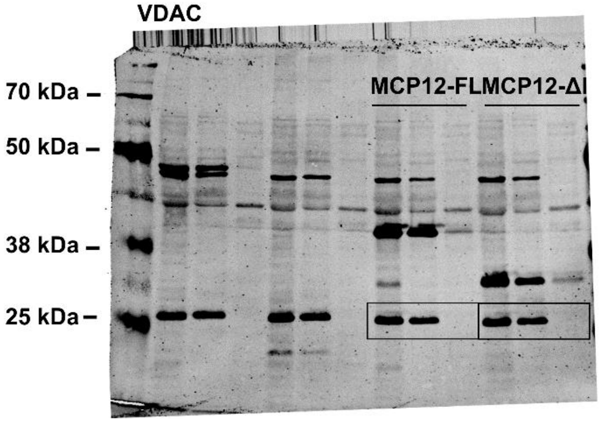

Licor - Chamaeleon Duo (P/N 928-60000)

Full scans for Figure 6c

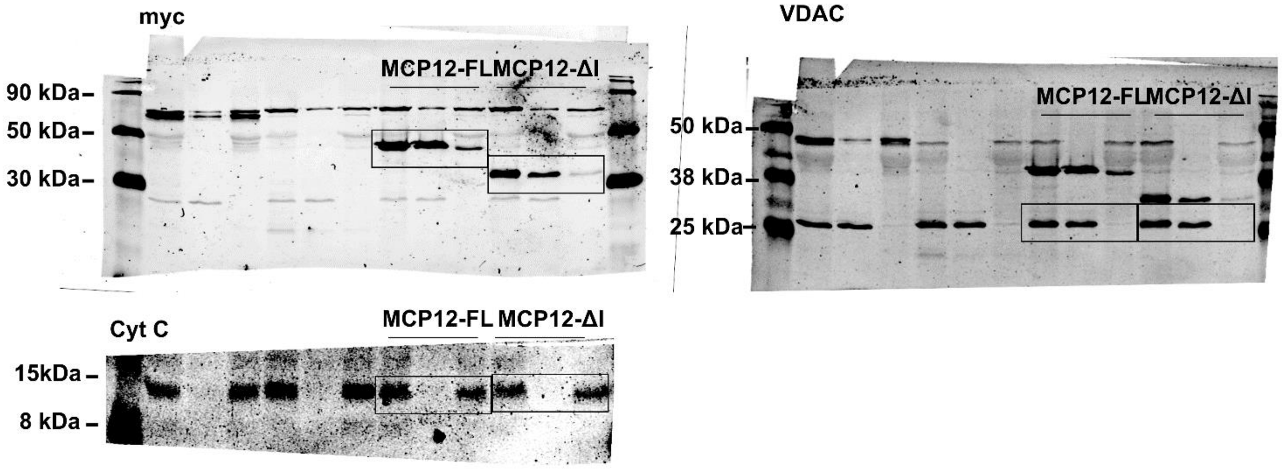

Licor - Chamaeleon Duo (P/N 928-60000)

Full scans for Figure 6d

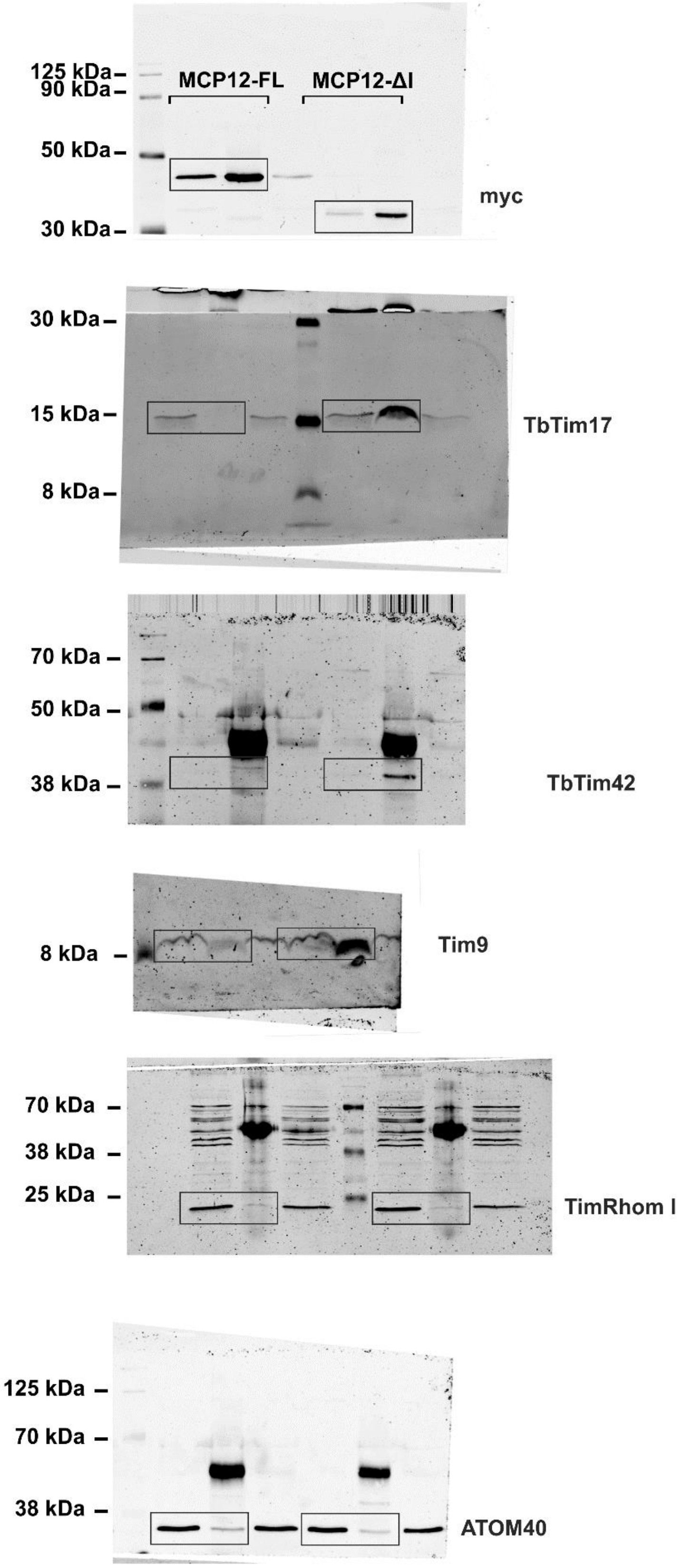

Licor - Chameleon Duo (P/N 928-60000)

Full scans for Supplementary Figure 1b

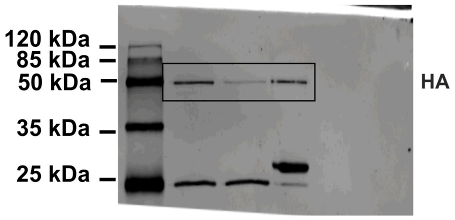

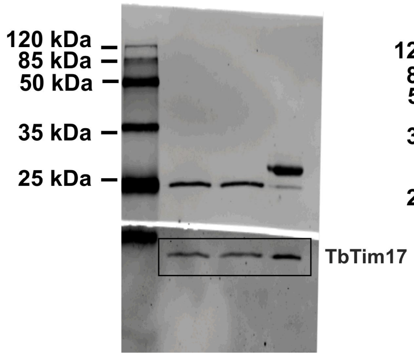

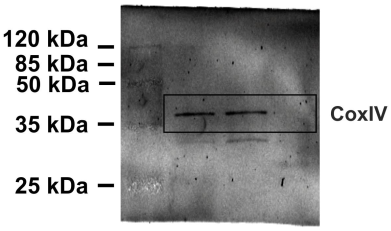

Thermo Scientific - Prestained protein molecular weight marker

Full scans for Supplementary Figure 1c

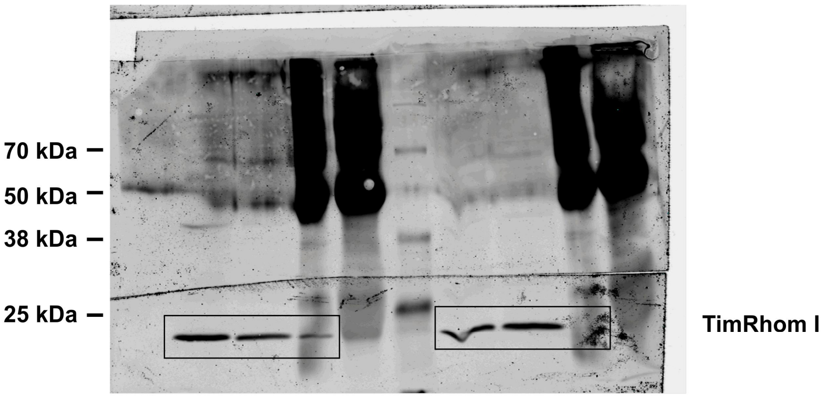

Licor - Chameleon Duo (P/N 928-60000)

TbTim17 (scanned at 680 nm)

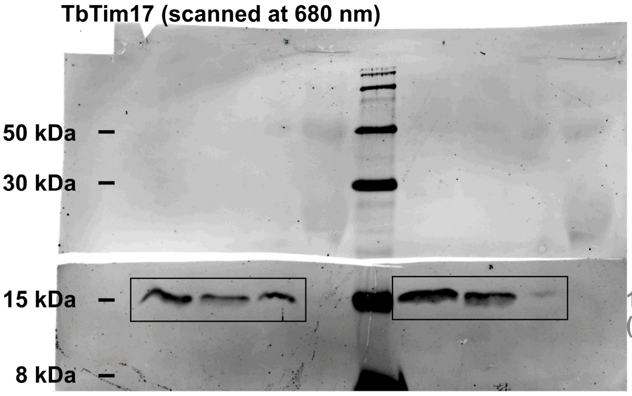

Cyt C (same blot as left: scanned at 800 nm)

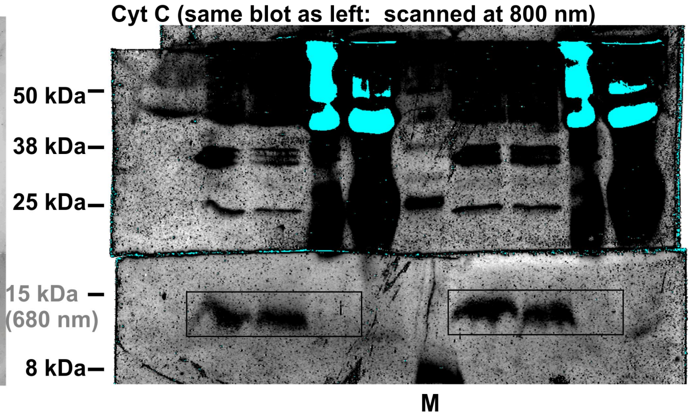

Licor - Chamaeleon Duo (P/N 928-60000)

Full scans for Supplementary Figure 4b

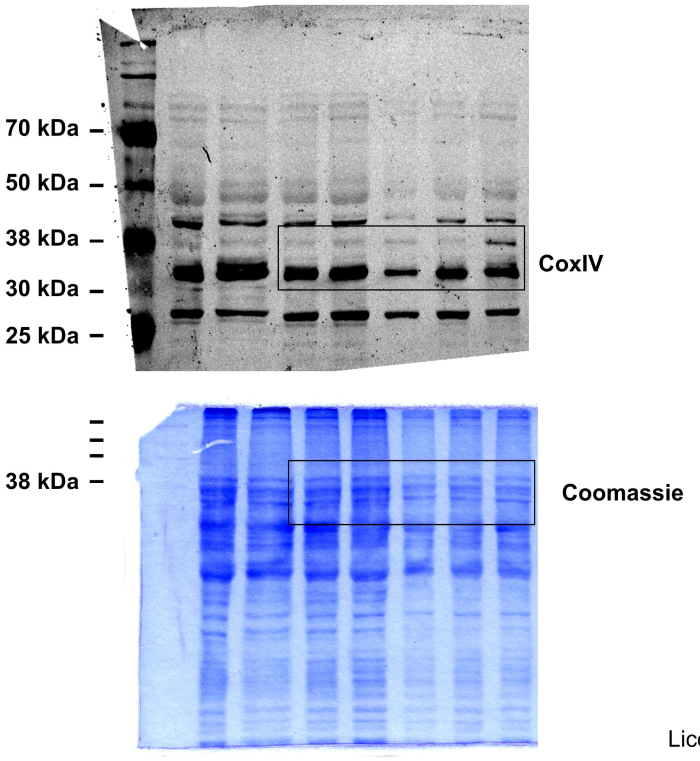

Licor - Chameleon Duo (P/N 928-60000)

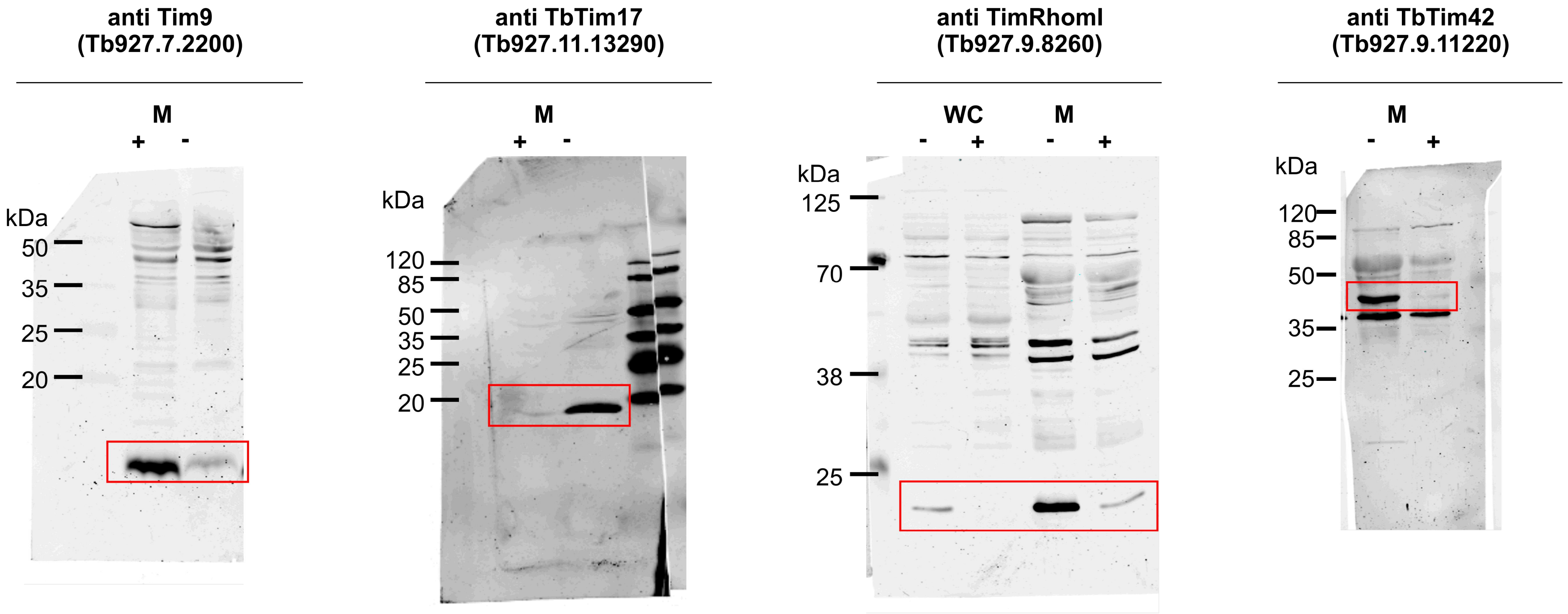

10 µg/lane of protein extract from purified mitochondria of a TbTim9 RNAi cell line (- = uninduced/ + = induced)

Thermo Scientific prestained Protein Molecular Weight Marker

10 µg/lane of protein extract from purified mitochondria of a TbTim17 RNAi cell line (- = uninduced/ + = induced)

Thermo Scientific prestained Protein Molecular Weight Marker

protein extract from whole cells (WC; $1.5 \cdot 10^6$ cells/lane) and purified mitochondria (M; 10 µg/lane) of uninduced (-) and induced (+) RNAi cell line targeting TimRhom I.

LI-COR Chameleon Duo prestained marker

protein extract from purified mitochondria (M; 10 µg/lane) of uninduced (-) and induced (+) RNAi cell line targeting TbTim42

LI-COR Chameleon Duo prestained marker

Supplementary Figure 5: Full scans and antibody information. Full scans of all blots (incl. molecular weight markers) shown in order of appearance and proof for specificity of newly prepared antibodies.

Supplementary Note 1: Primers used for production of the described cell lines

<u>TimRhom I ï HA-tagging</u>

forward	5ô- CATTATCTAGAATGTTACGGTACTCGCCGAT
reverse	5ô- GTATTGTCGACCAATGCTGCTGTAAGTTTGTC

<u>TbTim42 ï HA-tagging</u>

forward	5ô- CATTATCTAGAATGGCGTCGCGTTTGGCT
reverse	5ô- GTATTCTCGAGCAGAATCTCCACATCCCC

<u>TimRhom II ï myc-tagging</u>

forward	5ô- CATTAAAGCTTTCTAGAACCATGCTGCGGTTGCGGTGT
reverse	5ô- GTATTGGATCCCTCGAGCACGGCACCTGTGGTCAC

<u>TbTim13 ï HA-tagging</u>

forward	5ô- CATTATCTAGAATGCAACCCCCAACCCCA
reverse	5ô- GTATTCTCGAGGACCCCTCCCCCCTGCA

<u>TbTim17 ï myc-tagging</u>

forward	5ô- CATTAAAGCTTACCATGACAACACTTCTCGACCC
reverse	5ô- GTATTGGATCCGCGTTGAGCCAACCCCAATG

<u>mcp12 FL ï myc-tagging</u>

forward	5ô- CATTAAAGCTTTCTAGAACCATGTCGAAAGAGACAAAGGC
reverse	5ô- GTATTGGATCCCTCGAGCTGCTTTGCCTGAAGCTT

<u>mcp12 φ1 ï myc-tagging</u>

forward	5ô- CATTAAAGCTTTCTAGAACCATGGCCATCATGTCTCTGAAGG
reverse	5ô- GTATTGGATCCCTCGAGCTGCTTTGCCTGAAGCTT

<u>RNAi against TbTim17</u>

forward	5ô- CGCGGATCCAAGCTTATGACAACACTTCTCGACCC
reverse	5ô- GGGGTTGGCTCAACGCTAATCTAGACTCGAGCGG

<u>RNAi against TimRhom I</u>

forward	5ô- CATTAAAGCTTGGATCCGGTTACTTACCATTCCCTCC
reverse	5ô- GTATTTCTAGACTCGAGAATAAGACCACCACCAAGTG

<u>RNAi against TbTim42</u>

forward	5ô- CATTAAAGCTTGGATCCTTGTTGGCTACTTCAAGGAT
reverse	5ô- GTATTTCTAGACTCGAGCTGTAGGGCTTGAAGTCTTG

<u>RNAi against TimRhom II</u>

forward	5ô- CATTAAAGCTTGGATCCCACACCGAATTTGTTTGTGC
reverse	5ô- GTATTTCTAGACTCGAGCTGGGTGTTTCGGTAGCAAT

Supplementary References

1. Shannon, P. et al. Cytoscape: a software environment for integrated models of biomolecular interaction networks. *Genome Res.* **13**, 2498-504, (2003).
2. Pei, J., Kim, B.H. & Grishin, N.V. PROMALS3D: a tool for multiple protein sequence and structure alignments. *Nucleic Acids Res* **36**, 2295-300 (2008).
